# Using Objective Speech Analysis Techniques for the Clinical Diagnosis and Assessment of Speech Disorders in Patients with Multiple Sclerosis

**DOI:** 10.3390/brainsci14040384

**Published:** 2024-04-16

**Authors:** Zeynep Z. Sonkaya, Bilgin Özturk, Rıza Sonkaya, Esra Taskiran, Ömer Karadas

**Affiliations:** 1 Department of Experimental Linguistics, Ankara University, 06590 Ankara, Turkey; 2 Department of Neurology, Gülhane Medicine Faculty, Health Science University, 06010 Ankara, Turkey; drbilgin@gmail.com (B.Ö.); drrizasonkaya@gmail.com (R.S.); dromerkaradas@gmail.com (Ö.K.); 3 Department of Neurology, Antalya Training and Research Hospital, 07100 Antalya, Turkey; es_ranil@hotmail.com

**Keywords:** MS, speech disorders, quantitative speech analysis

## Abstract

Multiple sclerosis (MS) is one of the chronic and neurodegenerative diseases of the central nervous system (CNS). It generally affects motor, sensory, cerebellar, cognitive, and language functions. It is thought that identifying MS speech disorders using quantitative methods will make a significant contribution to physicians in the diagnosis and follow-up of MS patients. In this study, it was aimed to investigate the speech disorders of MS via objective speech analysis techniques. The study was conducted on 20 patients diagnosed with MS according to McDonald’s 2017 criteria and 20 healthy volunteers without any speech or voice pathology. Speech data obtained from patients and healthy individuals were analyzed with the PRAAT speech analysis program, and classification algorithms were tested to determine the most effective classifier in separating specific speech features of MS disease. As a result of the study, the K-nearest neighbor algorithm (K-NN) was found to be the most successful classifier (95%) in distinguishing pathological sounds which were seen in MS patients from those in healthy individuals. The findings obtained in our study can be considered as preliminary data to determine the voice characteristics of MS patients.

## 1. Introduction

Multiple sclerosis (MS) is a chronic, autoimmune, and demyelinating disease of the central nervous system that is commonly seen in young adults [1,2]. The disease affects several parts of the brain. It causes white matter lesions, leading to neurological impairments affecting motor, sensory, cerebellar, linguistic, and cognitive skills. As a matter of fact, during the past three decades, cognitive impairment has garnered more attention and research, and it is now commonly recognized as a basic component of MS, reducing physical independence and competence in activities of daily living [3]. Indeed, cognitive impairment increases morbidity in patients, is associated with a decrease in participation and functioning of daily life activities and appears to be associated with increased unemployment rates. In addition, findings of many studies have consistently demonstrated a pattern of decline in the following areas: ability to maintain attention over time, retrieving information received after time delay, information processing speed, visuospatial perception, abstraction ability, and language fluency [4].

Prevalence studies of community and clinical samples indicate that 53–65% of hospitalized MS patients develop cognitive impairments [5]. Also, the severity and type of cognitive impairment vary between individuals and can be observed in both early and progressive stages. The cognitive impairment, which is based on the findings of many studies, has consistently demonstrated a pattern of decline in language skills besides other cognitive and executive skills. However, most of the research that examined language deficits was carried out in English, and the assumptions made in English cannot be applied to other languages such as Turkish.

As it is well known, many patients have a range of language deficits, primarily dysarthria and loss of fluency, which affect the patient’s language production and reduce the quality of life. In the literature, studies suggest that speaking with plosive sounds without allowing the speech muscles to coordinate and stressing some words inappropriately are considered typical MS symptoms [1,2,3]. However, these linguistic deficiencies, which are regarded as cardinal symptoms of the disease, cannot be measured as objective parameters due to the fact that they cannot be determined using quantitative methods [5,6]. Moreover, these language deficits can be seen not only in MS, but also in some other neurodegenerative diseases, such as Parkinson’s disease and amyotrophic lateral sclerosis (ALS) [7,8,9].

Neurodegenerative disorders can impact different parameters of speech in different ways due to the involvement of various brain regions and the existence of different pathological processes such as gray or white matter damage or axonal or dendritic diseases. It is estimated that the determination of these parameters through quantitative methods can be used as a marker in the necessary diagnosis and distinctive diagnosis of diseases, as well as in the follow-up to treatment [2,3]. Indeed, the rapid development of technology and interdisciplinary studies in recent years, with the increasingly important use of objective voice analysis methods for language and speech disorders, has accelerated, and great progress has been made in diagnosis and treatment [6,7,8]. One of these methods is acoustic sound analysis. In particular, this method is more widely accepted as an alternative technique of diagnosis to auxiliary laboratory methods. This method of analysis is based on the numerical processing of the sound sign which allows for an early diagnosis of vocal disorders, an objective detection of vocal function disturbances, and an objective comparison of sound before and after surgery and pharmacological therapy. On the other hand, the vowel metric is another method that is widely used in objective sound analysis. In clinical practice, this method is often used to associate dysarthria conditions with pathological vocal signals. However, despite all these technological advances and interdisciplinary studies, there are a limited number of studies in the literature where speech and voice characteristics of MS patients are studied using objective voice analysis methods [5,8,9,10,11]. Therefore, this interdisciplinary study, including Neurology, Linguistics, and Computer Sciences, aims to develop a system that can help diagnosis in the clinical environment and evaluate pathological sounds by using objective voice analysis methods to determine and perceive the voice characteristics of MS patients and disease activity.

## 2. Materials and Methods

The study was conducted on 20 (7 male + 13 female) patients of chronological age between 18 and 60 years, who applied to the Neurology Polyclinic at Health Science University, Gülhane Educational and Research Hospital, diagnosed with MS according to McDonald’s 2017 diagnostic criteria [12], as well as 20 (10 male + 10 female) healthy individuals with no neurological or vocal pathology (Table 1).

All patients suffered from relapsing–remitting multiple sclerosis (RRMS). Patients with MS had no other co-existing neurological disorders. All participants were native speakers of Turkish, had a minimum of a high school diploma, were not bilingual, and had at least learned a second language to some degree or another. These data are important in light of the fact that previous studies and theories have shown that the control of more than one language affects the process of perception and production of speech sounds [13]. The patients’ most recent attacks had been 3 months prior to participation in the study, and their treatments were steroid-free. Patients with MS were initially contacted by a neurology polyclinic. Each participant possessed Turkish as their native tongue. It was stated that they did not have any documented history of speech therapy or substance abuse. Additionally, they reported not having used antipsychotic medication in the past or present, and did not utilize a hearing aid. In terms of age and gender, the healthy subjects were matched with the patient group. Healthy individuals had no neurological or vocal pathology.

The average age of patients in the MS group was 29.50 years, with a standard deviation (sd) of 8.47 years. The age range of these patients was between 21 and 53 years. The control group consisted of healthy individuals with a mean age of 25.15 years (sd = 7.10 years). The age range of the participants in this group was from 18 to 57 years. Patients with MS disability were evaluated using the Expanded Disability Status Scale (EDSS) during neurological examinations. The average EDSS score for the participants with MS was 2.67 (sd = 1.76), with a range of 2 to 4.

The demographic and clinical features of the participants are shown in Table 1.

The speech and voice symptoms experienced by individuals with MS are commonly referred to as dysarthria and dysphonia [1,2,13,14,15]. Dysphonia is an indicator of the disease, but it also plays an important role in tracking the development of the symptoms. Loudness, unclearness, harshness, drop, and tremor in the voice are characteristic indicators of dysphonia. These indicators can be determined by analyzing the various frequencies of the sound. Therefore, only during the examination were the data obtained from the patient’s speech transmitted to the digital environment in the form of audio recordings. It is thought that an early detection of the disease can be possible depending on the distinctive features of an individual’s speech in patients with MS. Due to this reason, this study conducted a speaking assessment for each individual, with an average duration of five minutes. To avoid the risk of audio distortions and modifications during the recording process, participants were kept at a distance of 15 cm from the dynamic microphone, and sound samples were obtained from each individual. When taking sound samples, it is first necessary to identify the correct words and sound groups. Indeed, the sounds the patient speaks can provide a distinctive finding in MS. Therefore, when choosing the words to be spoken, the constant sounds, sentences, and vocal groups consisting of the words that would be most comfortable to see the changes in voice were used in this study.

The data acquisition took place in a quiet clinical laboratory with a controlled acoustic setup. Each participant was given a 5 min speaking test. All participants were instructed to speak with the neurologist for five minutes about their life and background. They were also informed that the neurologist would only intervene if they began to struggle with their speech. Thus, the pragmatic language productions were almost undirected, with the participant having full freedom of speech. Whenever the participants stopped speaking for more than 5 s, the neurologist asked questions to encourage speech production in the participant. Open questions were preferred instead of closed questions that can be answered in a few words, so as to intervene as little as possible in the outputs of participants. In sum, the interference by the neurologist was kept as short as possible. Because of our study procedure based on a continuous and sustained pronunciation of the vowels /a/, /e/, /i/, and /u/, after the five-minute speaking test, the acquisition of a continuous and sustained pronunciation of the vowels /a/, /e/, /i/, and /u/ was also performed for 5 s. The acquisition took place inside a clinical laboratory with optimal acoustic setup and with the subjects sitting comfortably.

To mitigate the risk of voice distortion and alteration during the recording process, a distance of 15 cm was maintained between the dynamic microphone and each participant as voice samples were obtained. The vowel production of male and female speakers in both groups was consistent with the age distribution across the groups. Furthermore, considering the potential influence of gender differences on frequency measurements, frequencies were standardized for gender. “A set of voices from healthy individuals aged 18 to 57 years was obtained. Males and females were separated in both sets based on their vocal frequency differences”.

Our study procedure was based on a continuous and sustained pronunciation of the vowels /a/, /e/, /i/, and /u/ for five seconds. In order to assess the speech behavior of patients with MS and healthy subjects, vocal data were analyzed to identify potentially significant patterns [10].

The vocal signal was captured using a dynamic microphone. The signals were captured in the wav format and examined using PRAAT. As is well known, PRAAT is a highly effective software program used in the quantitative acoustic evaluation of sound quality [16]. The received audio recordings were divided into audio files according to the different audio groups in the content of the editor program, and the attributes were defined to ensure the highest classification success for each audio sample. In the attribute extraction phase, the parameters identified were the vowel sound; the amplitude change rate, which quantifies the magnitude of energy transmitted by a wave; the pitch change ratio (pitch changes work on a logarithmic scale); the silence degree; and the Teager energy average (this calculation primarily displays the frequency and instantaneous changes in the signal amplitude, which is extremely sensitive to minor variations). Fluctuating conversion coefficients and high-grade statistical parameters were calculated. Following the acquisition procedure, the vocal signal was analyzed utilizing both acoustic voice analysis and vowel metric analysis methodologies.

Acoustic voice analysis is a quantitative technique that can be employed for the purpose of diagnosing, monitoring, and assessing the progression of sound pathologies. This technique enables the obtaining numerical and visual data regarding human voice. So, it makes it easier to analyze the current sound status. Due to its repeatable features, this procedure provides the opportunity to conduct comparisons [17,18,19,20,21,22]. At present, the parameters that are most extensively utilized by physicians and cited in research are **Fundamental Frequency (F0),** which shows the cycle of the wave; ***Jitter***, which refers to the degree of frequency variability in a sound wave and is typically stated as a word that distinguishes the cycle of F0; ***Shimmer***, which is a quantification of the degree of variation in amplitude inside a sound wave; and ***Harmonics-to-Noise-Ratio (HNR)***, which quantifies the amount of additional interference present in human vocal signals caused by a leakage in the closure of the vocal folds during speech production [5,10,12,16]. 

**Figure 1:**
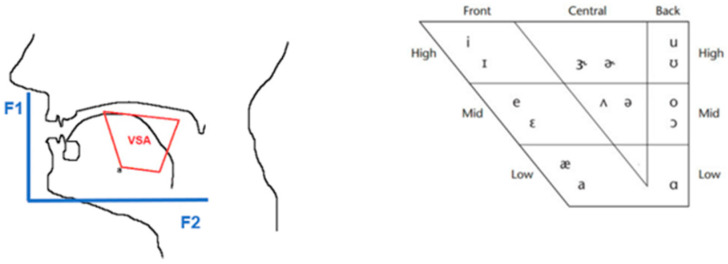
Vowel space area [10].

Vowel metric analysis is widely used in clinical research to associate dysarthria conditions with pathological vocal signals [10,11,23,24]. Speech in the presence of dysarthria is usually lower than articulative targets, and as a result, it is characterized by the centralization of vowels. Therefore, the vowel space area (VSA) index is calculated to assess the articulation of vocal sounds and consequently the centralization of these sounds. The VSA is a metric utilized to quantify the extent of the quadrilateral formed when the four corner vowels are projected onto the initial two formant frequencies (F1 and F2). It is frequently used to define the modifications made to speech motor control in order to provide the required explanation or clarification. High VSA values are suitable for healthy speech and hyper articulated vocal sounds. Low VSA is the result of the centralization of vocal sounds associated with pathological conditions. In dysarthric speech, the vocal signal is usually characterized by a decrease in VSA due to the centralization of the formant frequencies. Formants are distinct frequency peaks in the spectrum that exhibit a significant amount of energy and each of several prominent bands of frequency that determine the phonetic quality of a vowel. The VSA is therefore very useful for sound analysis and the monitoring of pathological sounds, as it allows for more detailed examinations. VSA calculations are performed using the well-known sound range method. The method is based on the presence of a speech signal related to the pronunciation of vocal sounds in both the acoustic and articulative spaces defined by the first two controls for vocal voices. Accordingly, the vocal sounds are in a two-dimensional space in the F1–F2 plane. The first controller defines the horizontal axis F1 and the second controller the F2 (Figure 1).

F1 is affected by the height of the tongue body and F2 by the front/back of the tongue body. In this articulative area, /u/, /a/, and /e/ are configured as the oscillating distances between the first and second controller coordinates for the corner vocals. Our study formulates a triangular VSA (tVSA) for calculating the first coordinate of the controller and a quadratic VSA (qVSA) for the second coordinate, in line with the findings of Sapir et al. (2010) and Fletcher et al. (2017), as reported in the literature [19,20].

As a result of acoustic analysis and the vowel metric analysis, 26 different attributes were identified from each sound sample (Table 2).

**Figure 2:**
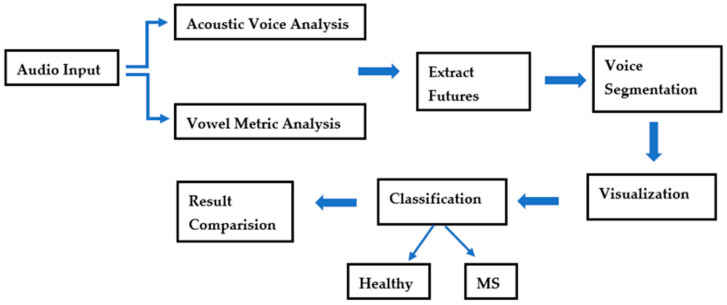
Block diagram used in study.

Accuracy, sensitivity, and specificity were measured using k-nearest neighbor (k-NN), support vector machines (SVM), and multi-layer perceptron (MLP) classification algorithms [25]. The leave-one-out test method was used to measure the success of the classifiers in distinguishing MS patients from the healthy subjects. In the final part, a similarity score was calculated by determining the most appropriate classification algorithm to determine whether the speaker has MS or not. The block diagram utilized in this study for diagnosing and assessing the voices of patients with MS is shown in Figure 2.

The subjects were recruited after providing signed consent forms prior to the trial. Before scheduling their participation, each stage of the research was verbally explained, and the participants were provided with information on the study protocol to verify their eligibility. Every participant was provided with a study information sheet to ensure they were well informed about the trial. Participants had the option to discontinue their involvement in the study at any given moment. The experimental protocol received approval from the Local Ethics Committee (Protocol No: 2023/11), and this study was performed strictly in accordance with the approved guidelines.

## 3. Results

In this interdisciplinary study, acoustic voice analysis and vowel metric analysis methods were used to distinguish between healthy and pathological voices. The mean values of the acoustic sound analysis results for patients and healthy individuals are shown in Table 3.

In our study, the mean F0 value of MS patients was found to be lower than that of healthy individuals. In terms of jitter values, the mean value was found to be less than 0.5% in healthy volunteers, whereas the patient group exhibited jitter values greater than 0.5%. The higher jitter values observed in patients with MS may be associated with a potential deficiency in controlling cord vibrations caused by the disease. Similarly, it was observed that the mean value of shimmer was higher in the MS patient group compared to healthy subjects. Also, the mean harmonic noise value was found to be higher in the MS patient group compared to healthy subjects. This indicates a decline in the speaking efficiency of patients with MS.

In order to achieve higher success in feature extraction in the block diagram designed in our study, vowel metric analysis was also performed, and the obtained data were calculated as tVSA and qVSA mean values for MS and healthy subjects (Table 4).

According to our results, the mean tVSA and qVSA values of MS patients showed a relative narrowing in the vocal ranges compared to healthy subjects. To facilitate a clearer comparison and verify the voice differences between the healthy and patient groups, our study devised an algorithm that maps F1/F2 pairs for each vocal sound on tVSA and qVSA graphs.

Moreover, a graphical presentation of qVSA values was created to verify the difference in the vocal sound fields of the MS patient group and healthy individuals. The qVSA was determined by measuring the area enclosed by the irregular quadrilateral created by the first and second formants (F1 and F2) of the corner vowels /a/, /u/, /e/, and /i/.

According to our findings, a reduction in the qVSA could be observed in patients with MS. This reduction represents a centralization of formant frequencies seen in dysarthria cases.

**Figure 3:**
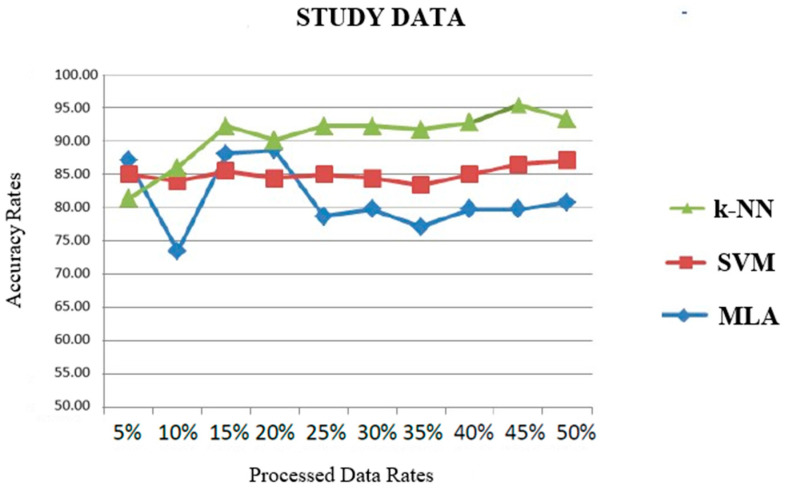
*Classifier sequences* in identifying MS disease voice features.

As a result of the acoustic voice and vowel metric analyses, 26 different attributes were determined for each sound in order to distinguish between healthy and pathological voices. The extracted features and the central tendency measures of these features were measured separately for accuracy, sensitivity, and specificity by using k-NN, SVM, and MLA classification algorithms [18,23,24]. The leave-one-out test method was used to measure the success of the classifiers in separating MS patients from the healthy subjects. The leave-one-out test is a cross-validation technique where one sample is excluded from the training set, and the remaining data are used to train the model [6,7]. In the final part, the most appropriate classifier algorithm was determined to estimate whether the speaker had MS or not. Our results showed that the k-NN method is the most suitable classifier for evaluating MS disease voice feature determination experiments with an accuracy rate of 95% (Figure 3).

**Figure 4:**
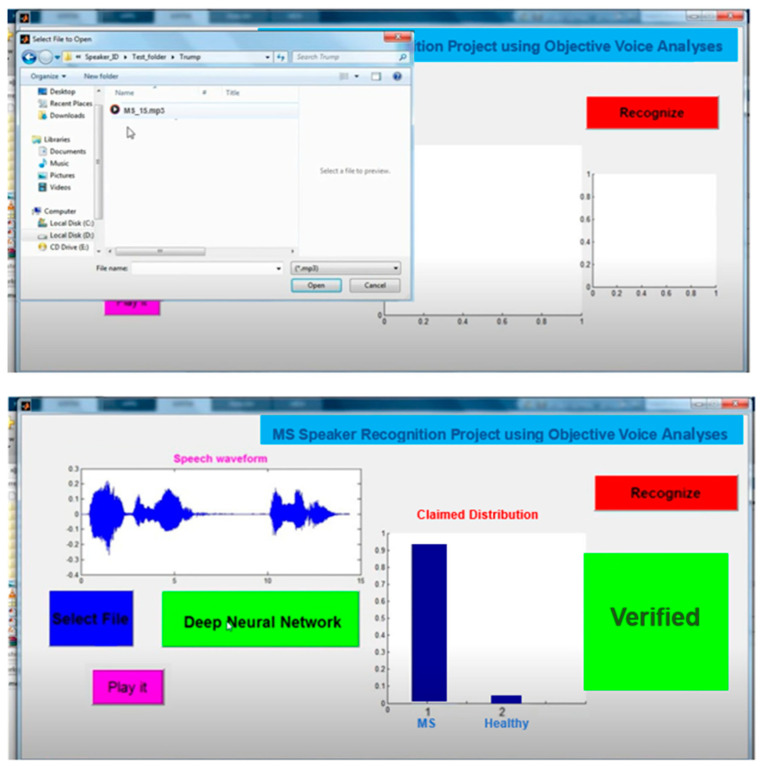
Developing voice recognition system in MS vs. healthy patients.

The extracted data were then loaded into a MATLAB software program, where further analysis could be conducted. Speaker voices were selected and transferred to the developed system. In the final part, the verification accuracy was observed, as well as whether the speaker had MS or not (Figure 4).

## 4. Discussion

The majority of patients with MS exhibit some language deficits, particularly dysarthria and a decrease in fluency [2]. As a matter of fact, in the literature, studies show that speaking with plosive sounds without allowing the speech muscles to coordinate and stressing some portions of words inappropriately can be considered one of the cardinal symptoms of MS [15,16]. Indeed, these evaluations are generally made with non-quantitative methods, which makes it impossible to objectively determine the deterioration in vocal function in the early stages of the disease [17]. However, with the rapid development of technology and interdisciplinary studies in recent years and the increasingly important use of objective voice analysis methods for language and speech disorders, voice changes can be detected in the early period, and if there is a pathological change, the degree of pathology and the mechanisms by which the current pathological situation occurs can be understood [9,13,18,19]. In fact, the investigation of language and speech disorders through the application of objective voice analysis techniques has grown in significance, and substantial advancements have been achieved in the identification, differentiation, and management of certain neurological disorders [11,14,20]. As a matter of fact, in a study conducted by Little et al., attributes related to Parkinson’s disease were identified by analyzing the vibration, tone, and volume of the voice. The researchers then assessed the level of dysphonia caused by the disease and its severity [25]. Similarly, in the study conducted by Lansford and Liss., the formant frequency values in the vocal sounds of Parkinson’s patients were examined, and it was concluded that the vowel triangle and vowel quadrilateral areas in the patients were narrowed. According to the authors, these measurements distinguish Parkinson’s patients from healthy individuals with an 80% success rate [26]. Also, Vashkevich and Rushkevich, used signal processing algorithms to determine the voice characteristics of early-stage ALS patients. The authors of a study that devised a disease-specific computer-based block system reported that the system distinguished ALS patients with an accuracy of 89% in clinical settings [27].

Despite the rapid advancement of technology and interdisciplinary studies, there is a limited number of studies in the literature where the speech and voice characteristics of MS patients are studied using objective voice analysis methods [9,10,21]. Similarly, the use of computer-based decision support systems for MS is not very common in clinical settings [5,16,22]. However, with objective voice analysis methods, changes in the voice can be detected even in the early stages of the disease and algorithms can be developed to distinguish whether the voices are pathological or healthy [4]. For this reason, in this interdisciplinary study, it was aimed to develop a system that can help diagnosis in the clinical environment and evaluate pathological sounds by using objective voice analysis methods to determine and perceive the voice characteristics of MS patients and disease activity. For this purpose, voice samples were taken from MS patients and healthy individuals. The vowel metric and acoustic sound analysis methods were used in our study and 26 different disease-specific attributes were determined. In the final part, appropriate classifier algorithms were created for these features and the success of distinguishing the voices of MS patients from healthy individuals was calculated with the created system. Our results showed that the k-NN method is the most appropriate classifier for distinguishing MS disease voice feature determination experiments with an accuracy rate of 95%. Similarly, in the study conducted by Ahmed et al., on Parkinson’s patients, it was stated that the k-NN classifier had a 97% success rate in determining whether the voice belonged to a Parkinson’s patient or a healthy individual [23].

An examination of voice signals revealed significant differences in vocal signal and vowel articulation between patients with RRMS and healthy individuals [2]. Specifically, studies have shown that individuals with MS exhibit a decrease in F0 and an increase in measures of vocal instability such as jitter, shimmer, and HNR when compared to individuals without MS [10,16]. Additionally, a decrease in VSA was seen in individuals with pathological diseases, both in terms of tVSA and qVSA. Our findings validate the initial hypothesis that patients with MS suffer from a speech disorder.

Two objective tests were used to validate the study results. According to our results significant differences could be found for the jitter and shimmer parameters in healthy subjects and patients with MS. Our study results have been clinically validated and can be regarded as an initial outcome for identifying pathological-voice-related parameters in MS patients. This finding could be useful in clinical applications for the early diagnosis and monitoring of MS disease.

## 5. Limitations

The findings obtained in our study can be considered as preliminary data to determine the voice characteristics of MS patients. However, our study has several limitations. The limited sample size might have prevented us from identifying all of the voice characteristics of MS. Another limitation of this study is that no evaluation could be made among MS subgroups. Future studies may concentrate on identifying new disease-specific sound parameters, exploring different classification methods, and using larger sample sizes with a wider range of severity. This is expected to significantly enhance the system’s performance and provide clinicians with an alternative diagnostic assessment method.

## Figures and Tables

**Table 1:** Demographic and clinical features of the participants.

	MS Patients (*n* = 20)			Healthy Subjects (*n* = 20)		
	Min	Max	(Mean ± sd)	Min	Max	(Mean ± sd)
Sex (m/f)	7/13			10/10		
Age	21	53	29.50 ± 8.47	18	57	25.15 ± 7.10
EDSS	2	4	2.67 ± 1.76			

**Table 2:** Attributes used in the study.

Jitter(ddp)	Frequency Perturbation Parameters	Shimmer(apq3)	Amplitude Perturbation Parameters	Mean Pitch	Pitch parameters
Jitter (ppg5)		Shimmer(apq5)		Median Pitch	
Jitter (local)		Shimmer(apq11)		Maximum pitch	
Jitter (local,absolute)		Shimmer(ddp)		Minimum pitch	
Jitter(rap)				Standard dev.	
Number of Period	Signal Parameters	Shimmer(local)	Articulation Parameters	F0	Prosodic parameters
Number of pulses		Shimmer (local,ddp)		F1	
Mean period		Fraction of locally		F2	
		Unvoiced frames			
		Number of voices			
		Breaks			

**Table 3:** Results for MS patients and healthy subjects.

	MS Patients			Healthy Subjects		
	Max	Mean	Min	Max	Mean	Min
F0	275.23	168.72	62.21	205.02	196.9	188.78
Jitter	1.71	0.9	0.09	0.4	0.32	0.25
Shimmer	17.10	8.87	2.98	6.90	5.83	4.76
Harmonic Noise Value (dB)	26.17	16.97	7.78	13.56	9.70	5.84

**Table 4:** The mean values of the vowel metric for MS and healthy subjects.

Metric	MS Patients	Healthy Subjects
tVSA	98.23	586.29
qVSA	109.57	689.34

## Data Availability

The data that support the findings of this study are available from the corresponding author upon reasonable request.
