# Correction: Sonkaya et al. Using Objective Speech Analysis Techniques for the Clinical Diagnosis and Assessment of Speech Disorders in Patients with Multiple Sclerosis. *Brain Sci.* 2024, *14*, 384

**DOI:** 10.3390/brainsci14101019

**Published:** 2024-10-14

**Authors:** Zeynep Z. Sonkaya, Bilgin Özturk, Rıza Sonkaya, Esra Taskiran, Ömer Karadas

**Affiliations:** 1 Department of Experimental Linguistics, Ankara University, 06590 Ankara, Turkey; 2 Department of Neurology, Gülhane Medicine Faculty, Health Science University, 06010 Ankara, Turkey; drbilgin@gmail.com (B.Ö.); drrizasonkaya@gmail.com (R.S.); dromerkaradas@gmail.com (Ö.K.); 3 Department of Neurology, Antalya Training and Research Hospital, 07100 Antalya, Turkey; es_ranil@hotmail.com

## Figure Updated

In the original publication [1], there was a mistake in the legend for Figures 1, 2 and 4. The figures were present, but the references were omitted. Also, some figures needed to be changed. In the original paper [1], Figures 1, 4 and 5 were completely omitted. Figure 1 was corrected (references were given), and Figure 2 and Figure 4 were added. The updated figures appear below:

**Figure 1:**
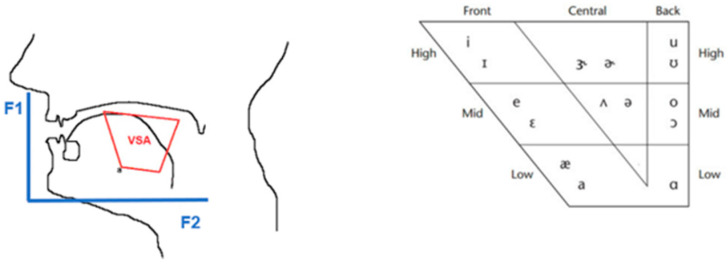
Vowel space area [10].

**Figure 2:**
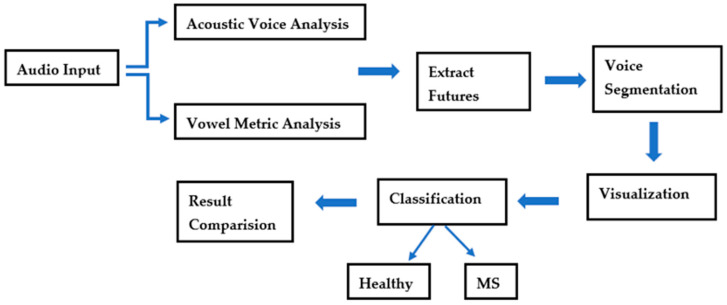
Block diagram used in study.

**Figure 4:**
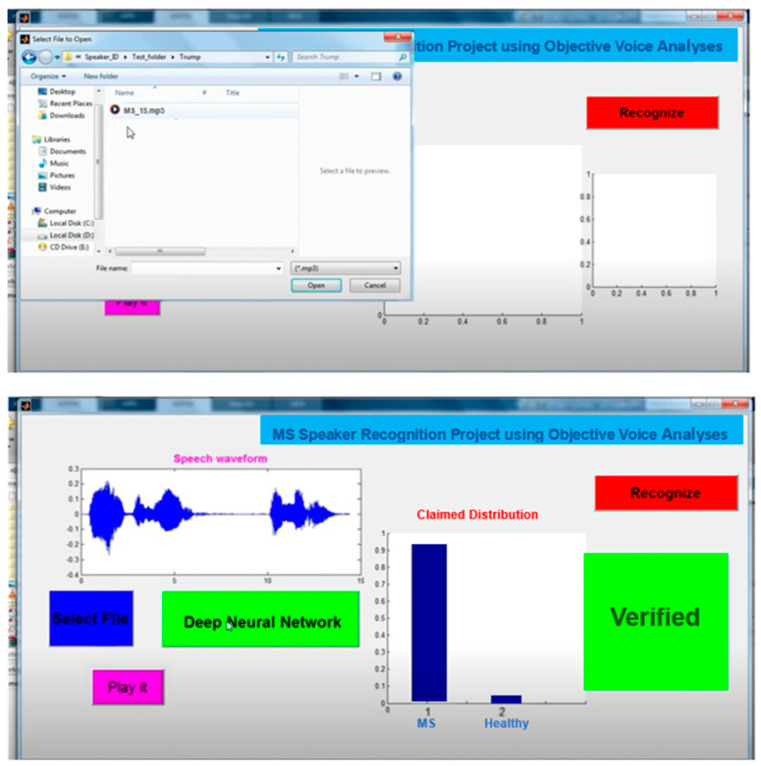
Developing voice recognition system in MS vs. healthy patients.

## Reference Update

In the original publication, ref [10] was not cited. With this correction, ref [10] has been added to the end of the eighth paragraph of Section 2, as well as to the caption Figure 1. The added ref [10] appears below:

10. Vizza, P.; Tradigo, G.; Mirarchi, D.; Bossio, R.B.; Veltri, P. On the Use of Voice Signals for Studying Sclerosis Disease. *Computers* **2017**, *6*, 30.

The original ref [10] has been change to ref [27], and the original refs [22,23] were removed. With this correction, the order of some references has been adjusted accordingly.

## Text Correction

A correction has been made to the first sentence in the ninth paragraph of Section 2. The updated sentence appears below:

The vocal signal was captured using a dynamic microphone. 

A correction has been made to the tenth paragraph of Section 2 to remove the mention of Figure 1. 

A correction has been made to the eleventh paragraph of Section 2, whereby the mention of Figure 2 has been updated to Figure 1. 

A correction has been made to the fourteenth paragraph of Section 2; the mention of Figure 3 has been updated to Figure 2. 

A correction has been made to the fourth paragraph of Section 3 to remove the mention of Figure 4. 

A correction has been made to the fifth paragraph of Section 3 to remove the mention of Figure 5. 

A new paragraph has been added to the end of Section 3. The contents appear below:

The extracted data were then loaded into a MATLAB software program, where further analysis could be conducted. Speaker voices were selected and transferred to the developed system. In the final part, the verification accuracy was observed, as well as whether the speaker had MS or not (Figure 4). 

The authors state that the scientific conclusions are unaffected. This correction was approved by the Academic Editor. The original publication has also been updated.
